# Relative Validity and Reproducibility of a Brief-Type Self-Administered Diet History Questionnaire for Japanese Children Aged 3–6 Years: Application of a Questionnaire Established for Adults in Preschool Children

**DOI:** 10.2188/jea.JE20140174

**Published:** 2015-05-05

**Authors:** Keiko Asakura, Megumi Haga, Satoshi Sasaki

**Affiliations:** 1 Interfaculty Initiative in Information Studies, the University of Tokyo, Tokyo, Japan; 1 東京大学大学院情報学環; 2 Department of Social and Preventive Epidemiology, School of Public Health, the University of Tokyo, Tokyo, Japan; 2 東京大学大学院医学系研究科 公共健康医学専攻 疫学保健学講座 社会予防疫学分野; 3 Research Center for Creative Partnerships, Ishinomaki Senshu University, Ishinomaki, Miyagi, Japan; 3 石巻専修大学共創研究センター

**Keywords:** validity, reproducibility, diet history questionnaire, Japanese, preschool children

## Abstract

**Background:**

Dietary intake assessment and subsequent dietary education or intervention in young children is important in decreasing prevalence of various noncontagious diseases in adulthood. Validation of diet assessment questionnaires for preschool children has just started in Japan. In this study, we rearranged the brief-type self-administered diet history questionnaire (BDHQ), a convenient diet assessment questionnaire that is widely used in a range of situations for adults, for use in children aged 3–6 years (BDHQ3y) and then validated the BDHQ3y in Japanese children.

**Methods:**

The guardians of 61 children aged 3–4 years completed the BDHQ3y twice at an interval of 1 month, along with a 3-nonconsecutive-day diet record (DR) between the two administrations of the BDHQ3y. Dietary intakes for energy and 42 selected nutrients were estimated using both the DR and the BDHQ3y. Mean intakes estimated by the two methods were compared, and correlation coefficients were calculated. Reproducibility of the BDHQ3y estimates was investigated using intra-class correlation coefficients (ICCs).

**Results:**

No significant differences in mean intakes estimated by the DR and the BDHQ3y were observed for one- to two-thirds of energy and examined nutrients. The median of Pearson correlation coefficients between intakes energy-adjusted by the residual method was 0.31 (interquartile range, 0.24 to 0.38). The median ICC was 0.72 (interquartile range, 0.63 to 0.76) for the crude nutrient intakes.

**Conclusions:**

Although the BDHQ3y might be a good candidate for dietary intake assessment in Japanese preschool children, its validity is currently moderate to low. Shortcomings should be overcome by obtaining and utilizing more information about children’s dietary habits.

## Abstract (ja)

**背景：:**

年少児における食事調査とそれに引き続く食育もしくは食事への介入は、成年後の様々な非感染性疾患を減少させるのに重要である。日本においては、就学前児童を対象とした食事調査用質問票の妥当性評価は始まったばかりである。本研究では、成人においては広範な目的で使用されている簡便な食事調査用質問票である簡易型自記式食事歴法質問票（BDHQ）を3－6歳の幼児用に改変（BDHQ3y）し、日本人幼児においてBDHQ3yの妥当性の評価を行った。

**方法：:**

3－4歳の幼児の保護者61人が1か月間隔で2回、BDHQ3yに回答した。また、その2回の調査の間の連続しない3日間において、食事記録法による食事調査が行われた。エネルギーと42の栄養素の摂取量が、食事記録法とBDHQ3yの両方で推定された。2つの方法で推定された平均摂取量が比較され、相関係数が計算された。BDHQ3yの推定値の再現性は、級内相関係数（ICC）を用いて検討された。

**結果：:**

食事記録法とBDHQ3yで推定された平均摂取量は、検討したうちの1/3～2/3のエネルギー・栄養素において有意差が認められなかった。残差法でエネルギー調整された摂取量についてのピアソン相関係数の中央値（四分位範囲）は、0.31（0.24-0.38）であった。粗摂取量のICCの中央値（四分位範囲）は0.72（0.63-0.76）であった。

**結論：:**

BDHQ3yは日本人就学前児童の食事調査に適切な質問票となりえるが、その妥当性は現時点では中程度～低いと言える。幼児の食習慣についてさらなる情報を得て質問票に反映させていくことで、欠点を克服してゆく必要がある。

## INTRODUCTION

Dietary intake assessment in young children is difficult for several reasons. Because young children aged under 7 or 8 years cannot remember and describe their dietary intake precisely, it must instead be reported by their guardians.^1^ However, the accuracy of parental reporting of children’s dietary intake is reported to be moderate.^2^ Further, estimation of food portion sizes usually consumed by young children is difficult.^1^ Estimation of consumed portion size is difficult even for adults, as evidenced by the various kinds of visual aids developed for this process, such as figures or food models.^3^ A second issue in portion size estimation is the increase in body size with growth: Japanese children aged 3–6 years typically grow approximately 6–7 cm and gain 2 kg per year.^4^^,^^5^ It is not certain that use of the same portion sizes is appropriate even in children measured at the same age.

Nevertheless, studies aimed at improving the dietary habits of children and investigating how dietary habits in childhood affect health in adulthood are important. The risk of cardiovascular disease and related conditions, such as hypertension and obesity, is closely related with dietary habits,^6^^,^^7^ and dietary habits are established in childhood and track into adulthood.^8^ Two different study groups have developed and validated food frequency questionnaires (FFQs) for young Japanese children.^9^^,^^10^ However, one of these validation studies reported the results of crude intake only.^9^ Further, as the study included children aged 3 to 11 years, the reported correlation might have been inflated by the spontaneous increase in the amount of food consumed with growth.^9^ Reporting for another FFQ was restricted to Spearman correlation coefficients for crude values of estimated nutrient intake.^10^

Sasaki et al have developed a comprehensive self-administered diet history questionnaire (DHQ)^11^ and a brief-type self-administered diet history questionnaire (BDHQ)^12^ for dietary intake assessment in adults. The validity of these questionnaires has been confirmed,^12^^–^^16^ and they are now widely used in epidemiological research.^17^^,^^18^ The BDHQ is a four-page questionnaire that takes about 15 minutes to complete and can be conveniently employed in field research involving a large number of subjects.

We recently reported food portion sizes of Japanese children aged 3–6 years, as estimated using a diet record (DR).^19^ This information enabled us to adjust the nutritional value calculation in the adult BDHQ to develop a new brief-type diet history questionnaire for Japanese preschool children aged 3–6 years (BDHQ3y).

In this study, we examined the relative validity and reproducibility of the BDHQ3y, which was developed using the BDHQ for adults as a model.

## METHODS

### Subjects

Apparently healthy children aged 3–4 years were recruited in Tome City, Miyagi Prefecture, from May to December, 2008. Children who were under guardian care at home (parental care in most cases) and who did not attend nursery school or kindergarten were invited to participate during attendance at municipal health centers for their three-year medical checkup, or at a childcare support center when they joined a play program. Exclusion criteria were: 1) presence of any disease or condition causing growth retardation; 2) current use of an elimination diet for food allergy; and 3) current use of other diet therapy for diseases such as diabetes mellitus, hyperthyroidism, or renal diseases. All guardians of children who met the criteria were asked to participate, and recruitment was continued until the number of subjects exceeded 60. This target number was determined based on the sample sizes of past studies.^10^^,^^20^^–^^22^ Ultimately, 61 children (31 boys and 30 girls) participated in the study. The participation rate of children was 48% (23 participated/48 invited) from childcare support centers and approximately 29% (38 participated/132 invited; invited number was estimated based on the number of target children for medical checkup in 2008) from municipal health centers. Researchers and research dietitians who were hired and trained for this study or were employees of the health promotion division of the Tome City Hall explained the aims and procedure of the study individually to all guardians of the participating children. All guardians gave written consent to participate in place of the children. The protocol of the study was approved by the Medical Ethics Committee of the Japan Society of Nutrition and Food Science (approval No. 70, approval date August 13, 2008).

### Study items and schedule

The surveys were implemented from June 2008 to February 2009. To standardize survey procedures, each research dietitian was provided with a detailed manual before the beginning of the study. The study schedule for each participating child was planned jointly by the guardian and the research dietitians. The duration of each survey was approximately one month. Initially, the guardian completed the BDHQ3y. They then completed a 3-nonconsecutive-day semi-weighed DR in the following month, followed by the BDHQ3y again. Body height and weight were reported in the BDHQ3y by the guardian, and values reported in the first BDHQ3y were used for analysis.

### Diet record

The three non-consecutive recording days for the DR consisted of two weekdays and one weekend day (Saturday or Sunday). The guardian with primary responsibility for food preparation for the subject (59 mothers and 2 grandmothers) recorded all foods and drinks consumed by the subject on the survey days. The main items recorded on the DR sheets were: 1) names of dishes and a simple graphic illustration; 2) names of foods and any ingredients in dishes; 3) whether the foods were home-made or ready-made; 4) approximate amount of consumed foods (estimated weight, amount measured by measuring spoon or measuring cup, or number of consumed foods [eg, two strawberries]); 5) measured weight of each dish (both prepared weight on the dish and leftover); and 6) place where the subject had the meal. The guardians were provided with a digital scale, measuring spoon, measuring cup, disposable camera, and graph paper ruled into 1-cm squares and were asked to complete all fields on the DR sheets. If a subject ate out and it was difficult to weigh dishes, the guardian took photos of the dishes on the graph paper to facilitate size estimation. The DR sheets for each survey day were directly handed to the research dietitian or mailed to the research center immediately after recording, and then checked by the research dieticians as soon as possible. If missing or unclear information was recorded on the sheet by the guardian, the research dietitian questioned the guardian directly or by phone or fax. After this confirmation process, food item numbers^23^ were assigned to all recorded foods and beverages, and, if necessary, consumed weight was estimated as precisely as possible. Since major causes of inaccurate intake estimation by the guardians (surrogate reporters of children) were omission of recording (eg, when a child consumed foods but his/her guardian was unaware) and misreporting of consumed amounts,^1^ immediate confirmation of the records and careful weight estimation was performed. Recorded food weights were then reconfirmed by at least two research dietitians, and nutritional value calculations were performed with SAS version 9.3 (SAS Institute, Cary, NC, USA).

### Brief-type self-administered diet history questionnaire for preschool children

The BDHQ3y was developed for assessment of dietary intake during the preceding month in Japanese preschool children aged 3–6 years. The BDHQ3y is a four-page structured questionnaire whose basic structure is almost the same as the BDHQ for adults, except for the exclusion of one section about alcoholic beverages. Thus, it includes four sections: (1) intake frequency of 57 food and non-alcoholic beverage items; (2) daily intake of rice, including type of rice (refined, unrefined, or rice boiled with barley), and miso soup; (3) usual cooking methods; and (4) general dietary behavior. For the BDHQ3y, items such as alcoholic beverages and coffee were excluded, while other items frequently consumed by children, such as yogurt drinks, french fries, chocolate, and ketchup, were included.

Daily intake of foods (66 items in total), energy, and selected nutrients were estimated using an ad hoc computer algorithm for the BDHQ3y. In the BDHQ3y, information on portion sizes was not collected; instead, an age-specific fixed portion size was used in calculations for all subjects. The same portion sizes were used for boys and girls of the same age in months, because boys and girls in this age range show no meaningful difference in basal metabolic rate (40 kcal/day for children aged 3–5 years and 60 kcal/day for children aged 6–7 years).^24^ Portion sizes of foods and beverages for the BHDQ3y were determined based on a previous study.^19^ In one study of dietary intake assessment in children, mothers more frequently underreported than overreported food intake.^2^ To adjust for the effect of underreporting, two possible causes of underreporting in dietary surveys using FFQ were considered: 1) omission of report on foods with small portion sizes; and 2) underreporting of intake frequency. The first is based on the observation that intake of ancillary items is more easily forgotten than intake of common foods or main course items^1^^,^^25^; in other words, average portion sizes of reported foods in the BDHQ3y are likely larger than those of all actually consumed foods. To account for this, the portion sizes of each food in the BDHQ3y were recalculated after eliminating those subjects with the lowest 10% consumption of that food from the calculation, using the same dataset as the previous study.^19^ Further, subjects might even forget reporting foods with large portion sizes. To adjust for this underreporting, nutritional value calculation was done using the same weighting factors as in the BDHQ.^12^

In addition, to account for physical growth and the increase in dietary intake in children, the nutritional value calculation also incorporated a weighting factor to adjust for the effect of age. We assumed that portion sizes increased 10% linearly for each 1-year increase in age for all kinds of foods^19^ and performed this adjustment using age in months.

### Data analysis

Crude values for the intake of energy and 42 selected nutrients were estimated based on the intake of food items obtained with the DR or BDHQ3y and the corresponding food composition list in the Standard Tables of Food Composition in Japan.^23^ Because intakes of most nutrients were positively associated with energy intake, energy-adjusted values were also calculated by the residual method using a regression model. Further, the density method was used to compute the amount of each nutrient consumed daily as a percentage from daily energy intake for energy-containing nutrients or per 1000 kcal of daily energy intake for non-energy-containing nutrients.^26^ All statistical analyses were performed on log-transformed values to account for non-normality.

Energy and nutrient intakes in the crude and energy-adjusted values for the DR (average values of DR in three days) and BDHQ3y (administered at the beginning of the study) were presented as means. Differences between values estimated by DR and BDHQ3y were tested with the paired *t*-test. Pearson and Spearman correlation coefficients between the values estimated by DR and BDHQ3y were then calculated. To evaluate the reproducibility of the BDHQ3y administered twice at an interval of one month, case 1 (assuming random selection of raters) intra-class correlation coefficients (ICCs) were computed using an SAS macro obtained from the SAS website (http://support.sas.com/documentation/onlinedoc/stat/ex_code/131/intracc.html).^27^

Finally, energy-adjusted food intakes estimated using the DR and BDHQ3y were presented as medians because distributions of the food intakes were highly skewed. Food groups were defined according to a previous study of the DHQ and the BDHQ3y.^12^ Differences between the values were tested with the Wilcoxon signed-rank test. Spearman correlation coefficients between food intakes measured using the DR and BDHQ3y were then calculated. All statistical analyses were performed using SAS version 9.3. Reported *P* values were two-tailed and considered statistically significant at the 0.05 level.

## RESULTS

The study participants are characterized in Table 1. Mean age, height, weight, and BMI did not significantly differ between boys and girls.

**Table 1.** Characteristics of study subjects

	Boys (*n* = 31)	Girls (*n* = 30)	Total (*n* = 61)
	Mean ± SD or *n* (%)	Mean ± SD or *n* (%)	Mean ± SD or *n* (%)
Age, years	3.3 ± 0.5	3.2 ± 0.4	3.3 ± 0.5
3 years	21 (67.7)	23 (76.7)	44 (72.1)
4 years	10 (32.3)	7 (23.3)	17 (27.9)
Body height, cm	98.8 ± 4.4	96.7 ± 5.1	97.8 ± 4.8
Body weight, kg	15.3 ± 1.6	14.9 ± 2.3	15.1 ± 2.0
BMI, kg/m^2^	15.6 ± 1.0	15.9 ± 1.8	15.7 ± 1.4

BMI, body mass index; SD, standard deviation.

BMI was calculated by dividing body weight (kg) by the square of height (m).

Age, body height, body weight, and BMI were not significantly different between boys and girls by *t* tests.

Means of energy and nutrient intake estimated by the DR and BDHQ3y are shown in Table 2. Energy intake estimated by the BDHQ3y was significantly underestimated compared to the DR (*P* = 0.0017, paired *t* test). Regarding the crude intakes of the 42 nutrients, no significant differences between the DR and BDHQ3y were observed for 19 (45%) nutrients. For energy-adjusted intake of nutrients by the residual method and the density method, no significant differences were observed for 15 (36%) nutrients and 25 (60%) nutrients, respectively. Pearson and Spearman correlation coefficients between nutrient intake values estimated by the DR and BDHQ3y are shown in Table 3. Correlation coefficients between the two estimated energy intakes were 0.18 (Pearson) and 0.22 (Spearman), and neither were statistically significant. Regarding the crude intake values of 42 nutrients, the median Pearson correlation coefficient was 0.26 (interquartile range [IQR], 0.17 to 0.35). Regarding the energy-adjusted values, the median Pearson correlation coefficient was 0.31 (IQR, 0.24 to 0.38) using the residual method and 0.30 (IQR, 0.23 to 0.36) using the density method. Most correlation coefficients for energy-adjusted nutrient intake values were improved compared with those for the crude values. The Pearson and Spearman correlation coefficients were similar. Reproducibility of the BDHQ3y was then assessed by calculating the ICC (Table 4). All values estimated by the second BDHQ3y were smaller than those estimated by the first BDHQ3y. The standard deviation (SD) values of nutrient intakes were smaller in the second survey, except for saturated fat, α-carotene, β-carotene, β-carotene equivalent, riboflavin, and calcium. The median ICC was 0.72 (IQR, 0.63 to 0.76) for the crude nutrient intakes, and 0.63 (IQR, 0.57 to 0.68) for the energy-adjusted nutrient intakes by the residual method and 0.63 (IQR, 0.56 to 0.69) for those by the density method.

**Table 2.** Mean energy and nutrition intakes estimated by diet records and BDHQ3y among 61 Japanese preschool children aged 3–4 years

	Crude and energy-adjusted by the residual method^a^						Energy adjusted by the density method^a^					
	
	Unit	DR		BDHQ3y		*P* value^b^	Unit	DR		BDHQ3y		*P* value^b^
			
		mean	SD	mean	SD			mean	SD	mean	SD	
Energy	(kcal/day)	1137.28	228.02	1018.75	233.14	—		—	—	—	—	
Protein	(g/day)	37.70	9.07	33.36	8.05	<0.01	(% energy)	13.26	1.69	13.17	1.68	0.75
Fat	(g/day)	36.10	11.00	33.56	8.57	0.02	(% energy)	28.48	5.84	29.69	3.64	0.05
Saturated fat	(g/day)	12.06	4.58	11.51	3.27	0.54	(% energy)	9.52	2.80	10.22	2.06	0.02
Monounsaturated fat	(g/day)	12.29	4.10	11.19	3.02	0.02	(% energy)	9.69	2.45	9.88	1.35	0.23
Polyunsaturated fat	(g/day)	6.52	2.03	7.03	2.06	0.03	(% energy)	5.16	1.20	6.19	1.01	<0.01
n-6 polyunsaturated fat	(g/day)	5.44	1.63	6.05	1.82	<0.01	(% energy)	4.31	0.99	5.31	0.88	<0.01
n-3 polyunsaturated fat	(g/day)	1.14	0.51	1.15	0.41	0.70	(% energy)	0.90	0.32	1.01	0.24	0.01
Marine-origin n-3 polyunsaturated fat^c^	(g/day)	0.34	0.33	0.29	0.18	0.44	(% energy)	0.26	0.22	0.26	0.14	0.85
Eicosapentaenoic acid	(g/day)	0.11	0.11	0.09	0.06	0.33	(% energy)	0.09	0.08	0.08	0.05	0.96
Docosahexaenoic acid	(g/day)	0.20	0.19	0.17	0.10	0.26	(% energy)	0.15	0.13	0.15	0.08	0.87
α-linolenic acid	(g/day)	0.76	0.27	0.83	0.28	0.06	(% energy)	0.60	0.19	0.73	0.16	<0.01
Cholesterol	(mg/day)	197.03	98.00	152.42	54.09	<0.01	(mg/1000 kcal)	174.51	80.52	150.93	49.21	0.16
Carbohydrate	(g/day)	162.61	34.44	143.27	35.85	<0.01	(% energy)	57.30	6.08	56.14	4.43	0.23
Total dietary fiber	(g/day)	6.40	2.37	5.58	2.05	<0.01	(g/1000 kcal)	5.59	1.70	5.43	1.40	0.57
Soluble dietary fiber	(g/day)	1.63	0.64	1.38	0.55	<0.01	(g/1000 kcal)	1.42	0.45	1.34	0.40	0.20
Insoluble dietary fiber	(g/day)	4.47	1.72	4.14	1.49	0.07	(g/1000 kcal)	3.91	1.28	4.03	1.00	0.25
Retinol	(µg/day)	133.15	64.66	163.35	57.25	<0.01	(µg/1000 kcal)	117.68	51.49	162.52	52.50	<0.01
Vitamin A (retinol equivalent)^d^	(µg/day)	269.05	103.33	256.48	93.20	0.38	(µg/1000 kcal)	240.23	91.98	253.74	79.66	0.16
α-carotene	(µg/day)	190.69	123.29	165.16	110.34	0.32	(µg/1000 kcal)	171.07	115.46	162.96	107.07	0.94
β-carotene	(µg/day)	1125.87	663.91	920.16	588.75	0.02	(µg/1000 kcal)	1007.24	613.47	896.66	542.52	0.27
β-carotene equivalent^e^	(µg/day)	1384.69	781.67	1111.25	657.46	0.03	(µg/1000 kcal)	1233.92	707.67	1088.25	606.16	0.31
Cryptoxanthin	(µg/day)	298.48	539.93	184.56	180.05	0.03	(µg/1000 kcal)	256.24	471.52	187.91	186.30	0.01
Vitamin D	(µg/day)	4.16	3.45	4.04	2.36	0.35	(µg/1000 kcal)	3.58	2.63	3.99	1.98	0.03
α-tocopherol	(mg/day)	3.68	1.10	3.43	0.99	0.05	(mg/1000 kcal)	3.26	0.81	3.37	0.62	0.23
Vitamin K	(µg/day)	87.39	61.26	98.27	57.37	0.15	(µg/1000 kcal)	76.23	49.42	94.81	48.60	<0.01
Thiamin	(mg/day)	0.51	0.14	0.39	0.09	<0.01	(mg/1000 kcal)	0.45	0.12	0.38	0.05	<0.01
Riboflavin	(mg/day)	0.88	0.41	0.71	0.18	<0.01	(mg/1000 kcal)	0.79	0.37	0.70	0.14	0.14
Niacin	(mg/day)	7.76	2.97	5.62	2.00	<0.01	(mg/1000 kcal)	6.93	2.71	5.53	1.43	<0.01
Vitamin B6	(mg/day)	0.60	0.20	0.52	0.16	<0.01	(mg/1000 kcal)	0.53	0.15	0.51	0.12	0.42
Vitamin B12	(µg/day)	4.01	2.42	3.01	1.51	<0.01	(µg/1000 kcal)	3.58	2.05	2.97	1.22	0.12
Folate	(µg/day)	145.86	55.09	121.37	40.69	<0.01	(µg/1000 kcal)	128.47	44.20	119.85	32.30	0.22
Pantothenic acid	(mg/day)	3.69	0.89	3.41	0.83	<0.01	(mg/1000 kcal)	3.26	0.53	3.37	0.51	0.11
Vitamin C	(mg/day)	72.60	74.81	45.52	21.67	<0.01	(mg/1000 kcal)	65.43	70.02	45.36	20.51	0.06
Sodium	(mg/day)	1764.39	562.89	2250.72	602.89	<0.01	(mg/1000 kcal)	1558.08	436.82	2223.03	402.36	<0.01
Potassium	(mg/day)	1339.75	347.79	1218.71	334.20	<0.01	(mg/1000 kcal)	1183.59	229.21	1202.62	227.79	0.57
Calcium	(mg/day)	398.61	147.20	399.79	121.15	0.68	(mg/1000 kcal)	355.74	124.78	396.60	103.20	<0.01
Magnesium	(mg/day)	129.38	35.94	125.61	33.41	0.21	(mg/1000 kcal)	113.71	21.03	123.29	17.96	<0.01
Phosphorus	(mg/day)	626.45	149.04	575.35	132.06	<0.01	(mg/1000 kcal)	553.48	88.76	569.32	80.33	0.16
Iron	(mg/day)	3.72	1.14	3.37	1.08	0.01	(mg/1000 kcal)	3.32	1.08	3.30	0.70	0.82
Zinc	(mg/day)	4.48	1.07	4.19	0.94	<0.01	(mg/1000 kcal)	3.96	0.63	4.14	0.37	0.02
Copper	(mg/day)	0.61	0.17	0.60	0.18	0.72	(mg/1000 kcal)	0.54	0.11	0.59	0.09	<0.01
Manganese	(mg/day)	1.34	0.42	1.49	0.47	0.01	(mg/1000 kcal)	1.18	0.31	1.46	0.35	<0.01

BDHQ3y, brief-type self-administered diet history questionnaire for preschool children; DR, diet record; SD, standard deviation.

The average values of DRs in three days and the values of the BDHQ3y implemented at the beginning of the study were compared in the table.

The means were shown as crude values or energy-adjusted values. Before statistical analysis, all variables were log-transformed.

^a^Energy adjustment was performed by the residual method and density method. Crude mean values and mean values obtained by residual method were the same.

^b^Two energy-adjusted mean values estimated using DR and BDHQ3y were compared by paired *t* tests. *P* values less than 0.05 indicate that the means estimated by DRs and BDHQ3y are significantly different.

^c^Sum of eicosapentaenoic acid, docosapentaenoic acid, and docosahexaenoic acid.

^d^Sum of retinol, β-carotene/12, α-carotene/24, and cryptoxanthin/24.

^e^Sum of β-carotene, α-carotene/2, and cryptoxanthin/2.

**Table 3.** Pearson and Spearman correlation coefficients between 3-day diet records and BDHQ3y for crude and energy-adjusted nutrient intakes and energy intake among 61 Japanese preschool children aged 3–4 years

	Crude		Energy-adjusted by the residual method		Energy-adjusted by the density method	
		
	Pearson	Spearman	Pearson	Spearman	Pearson	Spearman
Energy	0.18	0.22	—	—	—	—
Protein	0.26*	0.26*	0.33*	0.32*	0.32*	0.33*
Fat	0.08	0.03	0.27*	0.23	0.27*	0.21
Saturated fat	0.14	0.08	0.36*	0.41*	0.37*	0.41*
Monounsaturated fat	0.04	0.04	0.26*	0.10	0.25	0.11
Polyunsaturated fat	0.16	0.16	0.12	0.00	0.12	0.01
n-6 polyunsaturated fat	0.12	0.10	0.12	0.02	0.13	0.02
n-3 polyunsaturated fat	0.21	0.14	0.14	0.07	0.11	0.08
Marine-origin n-3^a^	0.43*	0.24	0.37*	0.22	0.31*	0.20
Eicosapentaenoic acid	0.43*	0.22	0.36*	0.20	0.29*	0.20
Docosahexaenoic acid	0.46*	0.25*	0.40*	0.22	0.35*	0.22
α-linolenic acid	0.10	0.04	0.13	0.09	0.13	0.08
Cholesterol	0.35*	0.36*	0.50*	0.51*	0.51*	0.47*
Carbohydrate	0.23	0.25*	0.27*	0.26*	0.27*	0.25
Total dietary fiber	0.33*	0.34*	0.45*	0.37*	0.44*	0.39*
Soluble dietary fiber	0.27*	0.32*	0.27*	0.27*	0.25*	0.28*
Insoluble dietary fiber	0.35*	0.36*	0.48*	0.44*	0.48*	0.44*
Retinol	0.26*	0.27*	0.30*	0.35*	0.30*	0.36*
Vitamin A (retinol equivalent)^b^	0.23	0.18	0.25	0.21	0.24	0.23
α-carotene	0.25	0.24	0.23	0.32*	0.23	0.30*
β-carotene	0.36*	0.26*	0.38*	0.38*	0.36*	0.34*
β-carotene equivalent^c^	0.33*	0.25*	0.38*	0.37*	0.36*	0.36*
Cryptoxanthin	0.32*	0.27*	0.34*	0.31*	0.34*	0.32*
Vitamin D	0.37*	0.24	0.35*	0.30*	0.34*	0.29*
α-tocopherol	0.14	0.16	0.20	0.08	0.20	0.10
Vitamin K	0.45*	0.46*	0.40*	0.33*	0.43*	0.39*
Thiamin	0.00	0.04	−0.16	−0.13	−0.12	−0.04
Riboflavin	0.17	0.23	0.12	0.32*	0.11	0.31*
Niacin	0.22	0.12	0.13	0.10	0.10	0.12
Vitamin B6	0.17	0.16	0.18	0.20	0.20	0.23
Vitamin B12	0.33*	0.19	0.30*	0.20	0.25	0.17
Folate	0.24	0.32*	0.33*	0.33*	0.34*	0.33*
Pantothenic acid	0.24	0.26*	0.39*	0.41*	0.39*	0.37*
Vitamin C	0.21	0.25	0.27*	0.32*	0.29*	0.31*
Sodium	0.35*	0.34*	0.17	0.12	0.13	0.07
Potassium	0.15	0.21	0.25	0.28*	0.26*	0.28*
Calcium	0.38*	0.37*	0.45*	0.43*	0.43*	0.45*
Magnesium	0.26*	0.28*	0.36*	0.30*	0.36*	0.31*
Phosphorus	0.21	0.26*	0.38*	0.40*	0.39*	0.41*
Iron	0.39*	0.43*	0.32*	0.30*	0.32*	0.37*
Zinc	0.28*	0.29*	0.33*	0.30*	0.30*	0.28*
Copper	0.35*	0.35*	0.52*	0.44*	0.56*	0.53*
Manganese	0.30*	0.29*	0.31*	0.31*	0.33*	0.33*

BDHQ3y, brief-type self-administered diet history questionnaire for preschool children.

The average values of DRs in three days and the values of the BDHQ3y implemented at the beginning of the study were used.

Log-transformed energy and nutrient intake values were used for the calculation of correlation coefficient.

*P < 0.05: “*” means that there is statistically significant correlation between the values estimated by diet records and those estimated by the BDHQ3y.

^a^Sum of eicosapentaenoic acid, docosapentaenoic acid, and docosahexaenoic acid.

^b^Sum of retinol, β-carotene/12, α-carotene/24, and cryptoxanthin/24.

^c^Sum of β-carotene, α-carotene/2, and cryptoxanthin/2.

**Table 4.** Intra-class correlation coefficients between two BDHQ3y implemented with an interval of one month for crude and energy-adjusted nutrient intakes and energy intake among 61 Japanese preschool children aged 3–4 years

Nutrients	Unit	BDHQ3y_1^a^		BDHQ3y_2^a^		ICC		
		
		mean	SD	mean	SD	Crude	Adjusted 1(R)^b^	Adjusted 2(D)^b^
Energy	kcal/day	1018.75	233.14	984.66	219.12	0.73	—	—
Protein	g/day	33.36	8.05	32.15	6.90	0.72	0.62	0.61
Fat	g/day	33.56	8.57	32.18	7.64	0.70	0.70	0.71
Saturated fat	g/day	11.51	3.27	11.23	3.42	0.61	0.62	0.64
Monounsaturated fat	g/day	11.19	3.02	10.68	2.64	0.72	0.72	0.72
Polyunsaturated fat	g/day	7.03	2.06	6.65	1.73	0.76	0.60	0.60
n-6 polyunsaturated fat	g/day	6.05	1.82	5.72	1.51	0.77	0.59	0.59
n-3 polyunsaturated fat	g/day	1.15	0.41	1.09	0.32	0.70	0.55	0.52
Marine-origin n-3^c^	g/day	0.29	0.18	0.27	0.14	0.60	0.56	0.52
Eicosapentaenoic acid	g/day	0.09	0.06	0.09	0.05	0.58	0.54	0.50
Docosahexaenoic acid	g/day	0.17	0.10	0.15	0.08	0.61	0.57	0.56
α-linolenic acid	g/day	0.83	0.28	0.80	0.22	0.72	0.54	0.53
Cholesterol	mg/day	152.42	54.09	143.65	49.06	0.80	0.80	0.79
Carbohydrate	g/day	143.27	35.85	138.99	35.06	0.73	0.68	0.69
Total dietary fiber	g/day	5.58	2.05	5.24	1.88	0.81	0.75	0.77
Soluble dietary fiber	g/day	1.38	0.55	1.28	0.49	0.78	0.74	0.75
Insoluble dietary fiber	g/day	4.14	1.49	3.90	1.37	0.81	0.74	0.77
Retinol	µg/day	163.35	57.25	155.91	56.82	0.52	0.48	0.49
Vitamin A (retinol equivalent)^d^	µg/day	256.48	93.20	246.37	92.67	0.58	0.54	0.54
α-carotene	µg/day	165.16	110.34	164.26	118.01	0.63	0.64	0.63
β-carotene	µg/day	920.16	588.75	888.68	640.95	0.66	0.65	0.64
β-carotene equivalent^e^	µg/day	1111.25	657.46	1080.84	720.39	0.67	0.66	0.66
Cryptoxanthin	µg/day	184.56	180.05	187.22	177.62	0.60	0.56	0.56
Vitamin D	µg/day	4.04	2.36	3.72	1.65	0.61	0.52	0.50
α-tocopherol	mg/day	3.43	0.99	3.23	0.88	0.76	0.69	0.70
Vitamin K	µg/day	98.27	57.37	91.70	47.62	0.71	0.66	0.67
Thiamin	mg/day	0.39	0.09	0.38	0.08	0.72	0.63	0.57
Riboflavin	mg/day	0.71	0.18	0.70	0.20	0.56	0.49	0.56
Niacin	mg/day	5.62	2.00	5.27	1.56	0.73	0.61	0.59
Vitamin B6	mg/day	0.52	0.16	0.49	0.13	0.76	0.71	0.69
Vitamin B12	µg/day	3.01	1.51	2.87	1.10	0.70	0.66	0.64
Folate	µg/day	121.37	40.69	115.03	38.73	0.73	0.64	0.64
Pantothenic acid	mg/day	3.41	0.83	3.33	0.80	0.64	0.57	0.58
Vitamin C	mg/day	45.52	21.67	43.95	21.56	0.70	0.64	0.63
Sodium	mg/day	2389.75	606.73	2273.21	542.56	0.76	0.56	0.55
Potassium	mg/day	1219.06	334.21	1184.11	326.53	0.72	0.62	0.62
Calcium	mg/day	399.87	121.14	398.47	141.85	0.62	0.62	0.63
Magnesium	mg/day	125.67	33.41	120.49	30.73	0.77	0.58	0.58
Phosphorus	mg/day	575.35	132.06	559.61	130.95	0.68	0.61	0.62
Iron	mg/day	3.37	1.08	3.19	0.91	0.82	0.70	0.71
Zinc	mg/day	4.19	0.94	4.08	0.86	0.74	0.63	0.62
Copper	mg/day	0.60	0.18	0.58	0.16	0.82	0.67	0.69
Manganese	mg/day	1.49	0.47	1.43	0.44	0.77	0.72	0.72

BDHQ3y, brief-type self-administered diet history questionnaire for preschool children; ICC, intra-class correlation coefficient (Case 1); SD, standard deviation.

Log-transformed energy and nutrient intake values were used for the calculation of intraclass correlation coefficient.

^a^The column for BDHQ3y_1 shows the crude energy and nutrient intake values estimated by the BDHQ3y implemented at the beginning of the study period. That for BDHQ3y_2 shows those estimated by the BDHQ3y implemented at the end of the study period.

^b^Adjusted 1(R) shows the energy-adjusted nutrient intake values by the residual method. Adjusted 2(D) shows those adjusted by the density method.

^c^Sum of eicosapentaenoic acid, docosapentaenoic acid, and docosahexaenoic acid.

^d^Sum of retinol, β-carotene/12, α-carotene/24, and cryptoxanthin/24.

^e^Sum of β-carotene, α-carotene/2, and cryptoxanthin/2.

The comparison between energy-adjusted food intakes estimated by the DR and the BDHQ3y is shown in Table 5. Intakes of eight food groups (40% in 20 groups) estimated using the BDHQ3y were significantly different from those estimated using the DR. Significant correlation between the estimated intakes was observed in 16 food groups (80%).

**Table 5.** Comparison of energy-adjusted food group intakes (g/1000 kcal) estimated by diet records and BDHQ3y among 61 Japanese preschool children aged 3–4 years

Food (g/1000 kcal)^a^	DR		BDHQ3y		Spearman correlation coefficient
	
	median	IQR	median	IQR	
Cereals	213	172–250	192	163–226	0.32*^c^
Rice	127	93–170	140	109–184	0.31*
Noodles	39.4	17.3–66.9	30.1*^b^	22.1–45.7	0.47*
Bread	9.4	0.0–22.9	10.7	6.0–21.4	0.50*
Beans	16.2	9.5–23.0	21.4*	12.8–31.0	0.42*
Potatoes	24.4	8.3–37.2	26.0	12.8–37.3	−0.10
Fruits	42.4	18.7–77.1	32.7*	10.9–67.5	0.51*
Vegetables^d^	69.0	41.3–96.2	58.5*	35.0–79.4	0.34*
Green and yellow vegetables^d^	15.5	6.3–28.1	14.6	7.5–27.0	0.49*
Other vegetables^d^	33.3	23.4–50.9	30.6*	16.7–42.0	0.13
Fish and Shellfish	23.8	13.1–28.9	20.3	16.9–31.6	0.25
Meat	28.8	18.7–38.6	24.0	19.0–28.7	0.11
Eggs	14.3	5.2–24.1	11.1*	7.7–18.7	0.45*
Dairy products^e^	129	84–207	130	89–247	0.56*
Confectioneries	42.0	23.7–69.4	59.0*	37.8–77.3	0.34*
Fat and oil	6.4	4.4–8.7	6.7	5.6–8.7	0.27*
Beverages^f^	223	130–372	183	108–306	0.49*
Fruit and vegetable juice	14.7	0.0–64.5	12.8	4.6–49.7	0.43*
Tea^g^	105	45–259	78*	32–193	0.59*
Soft drinks^h^	49.7	0.0–113.3	40.3	19.3–84.4	0.43*

BDHQ3y, brief-type self-administered diet history questionnaire for preschool children; DR, diet record; IQR, interquartile range.

The average food intakes of DRs in three days and the values of the BDHQ3y implemented at the beginning of the study were compared in the table.

^a^Food intakes are shown as grams per 1000 kcal energy intake.

^b^Two energy-adjusted food intakes estimated using DR and BDHQ3y were compared by Wilcoxon signed-rank tests. *P < 0.05: “*” means that there is statistically significant difference between the two estimated intakes.

^c^*P < 0.05: “*” means that there is statistically significant correlation between the values estimated by DR and BDHQ3y.

^d^The category of “Vegetables” included green and yellow vegetables, other vegetables, pickled vegetables, mushrooms, and seaweeds. Green and yellow vegetables and other vegetables were classified based on a notification from the Ministry of Health, Labour and Welfare.

^e^Total dairy products intake except for ice cream and lactic acid bacteria beverages.

^f^All beverages except for water.

^g^All kinds of teas.

^h^Lactic acid bacteria beverages, fruit juice excluding 100% juice, cocoa, cola and sugar-sweetened soft drinks (including sports drinks).

## DISCUSSION

Many previous studies have reported that FFQs overestimate energy and nutrient intake in children compared with the reference method, such as DR or 24-hour recall.^20^^,^^21^^,^^28^^,^^29^ These studies attempted to apply FFQs developed for adults to children and discussed the necessity of adequate adjustment for portion sizes.

Our present results indicated that the BDHQ3y tends to underestimate mean energy intake and some nutrient intakes. We calculated portion sizes for the BDHQ3y after eliminating the 10% of subjects with the lowest consumption for all individual foods in the analysis of another DR.^19^ Although the cutoff point of 10% appeared suitable in eliminating the intake of small portion sizes, such as parmesan cheese on pasta or small pieces of ham in salad, whose reporting was easily forgotten, it is unclear whether this cutoff point is appropriate. To date, no studies have quantitatively evaluated the recognition and memory of foods in small quantities. Underestimated intakes included energy, protein, carbohydrate, dietary fiber, and several water-soluble vitamins. Since mean intake values after energy adjustment by the density method did not differ for protein and carbohydrate, the portion sizes of foods consisting mainly of energy-providing nutrients should be increased in the nutritional value calculation of the BDHQ3y. In fact, the medians estimated by the BDHQ3y for noodles, fruits, and eggs were significantly lower than those by the DR. In addition, we were unable to obtain sufficient data about the portion sizes of snacks in the analysis of the other DR.^19^ In further validation studies in other populations, information about snacks in the DR analyzed in this study can be used to adjust the nutritional value calculation in the BDHQ3y.

Although the correlation coefficients for cholesterol, dietary fiber, and some minerals were fairly good, they were clearly low for mono- and polyunsaturated fat and some water-soluble vitamins. The BDHQ3y partially uses the diet history method to estimate the intake of some nutrients. For example, the amount of cooking oil used for meal preparation is estimated by utilizing information about the usual cooking method for each subject. However, detailed information about the type and amount of cooking oil typically used in each meal preparation process has not been reported before. This uncertainty might have affected the correlation coefficients not only for fatty acids but also for α-tocopherol, which is abundant in cooking oils. Regarding water-soluble vitamins, the correlation coefficients for thiamin, niacin, and vitamin B_6_ were particularly low, whereas those for folate, pantothenic acid, and vitamin C were relatively high. The Japanese population generally obtains the first three B vitamins from a variety of foods, among which animal meats are major contributors.^30^^,^^31^ In contrast, the major sources of folate, pantothenic acid, and vitamin C are vegetables or cereals.^30^^,^^31^ It is possible that the intake of some vitamins was not correctly estimated due to inaccurate portion sizes or an inappropriate food list for meats. This assumption might be supported by the relatively low correlation between the estimated intakes of meat by the DR and the BDHQ3y.

Generally speaking, the FFQ has lower validity for children, particularly those aged under 12 years, than for adults.^32^ In the validation studies for adults, average correlations for energy-adjusted nutrients have generally ranged from 0.45 to 0.7,^32^ but these correlations were lower in the FFQ for children. For example, Fumagalli et al reported Pearson correlation coefficients of between 0.12 and 0.45 for energy-adjusted nutrients in their study of the FFQ for 5- to 10-year-old children.^28^ Blum et al reported relatively good correlation coefficients between 0.26 and 0.63 for their FFQ for 3- to 5-year-old children.^33^ In contrast, Stein et al reported sex-segregated correlation coefficients for 12 energy-adjusted nutrients (24 coefficients in total), of which 17 were lower than 0.3.^29^ Compared with these other studies, the BDHQ3y appears to be a potentially useful tool for dietary intake assessment in Japanese preschool children.

Reproducibility of the BDHQ3y was considered to be good.^34^^,^^35^ However, the one-month interval might have been too short to evaluate reproducibility, because subjects might remember their answer to the first questionnaire, and a high ICC might merely reflect this memory. Many FFQ validation studies evaluated reproducibility with a one-year interval.^32^ Our present subjects were preschool children, so rapid dietary intake change owing to growth had to be taken into account. We also observed smaller mean and SD values of nutrient intakes in the second survey. These smaller values might have resulted from cognitive and behavioral change of the respondents after the first survey, such as more attention to dietary intake, which could have decreased reproducibility.

Several limitations of the present study warrant mention. First, the number of subjects was small (*n* = 61). However, a number of validation studies for FFQs included a similar number of subjects.^10^^,^^20^^–^^22^ Since the number of subjects necessary to detect a correlation coefficient of 0.35 with a significance level equal to 0.05 and with statistical power of 0.8 was estimated at 62 by nQuery Advisor R 7.0 (Statistical Solutions Ltd., Cork, Ireland), the number of subjects in our study was considered to meet the minimum requirement for this validation study. Second, subjects were limited to age 3 to 4 years, all living in a single area. Additional studies which include 5- to 6-year-old children living in this area as well as preschool children living in other areas in Japan should be conducted. Although we assumed for the BDHQ3y that portion sizes increase linearly with age, this should be confirmed based on actual data. Also, as all children included in this study were cared for at home, they usually had lunch at home, and their physical activity might have been different from those who attended nursery school or kindergarten. Therefore, generalizability of the results is limited. Third, the study subjects were not selected randomly but rather were volunteers. It is therefore likely that the subjects and their guardians were health-conscious and that their dietary habits differed from the general population. Compared to national data,^4^ the body constitution of the subjects in the present study might have been larger than the general population. Fourth, we used a 3-day DR as the reference standard for dietary intake. Although the reference should reflect “usual dietary intake”, a study in adults showed that estimating usual intake with 95% confidence intervals with deviation within 20% required measurement for more than 3 days for almost all nutrients.^36^ While longer-term DR are clearly more appropriate for reference, the considerable burden of recording on the subjects and their guardians had to be considered. In addition, several studies have used biomarkers, such as urinary excreted sodium,^32^ doubly-labelled water,^37^ or erythrocyte cell membrane fatty acid,^38^ as reference. They should be considered in future validation studies of BDHQ3y. Finally, the effect of seasonality on the validity of BDHQ3y was not considered in this study. The estimated nutrition intake in one season should not be interpreted as yearly average intake at this time.^39^

In conclusion, while the BDHQ3y might be a good candidate for dietary intake assessment in preschool children, the present study has identified a number of shortcomings requiring improvement. Efforts to enhance the validity of the BDHQ3y should be continued.

## ONLINE ONLY MATERIAL

Abstract in Japanese.
