# Supplementary material for: Relative Validity and Reproducibility of a Brief-Type Self-Administered Diet History Questionnaire for Japanese Children Aged 3–6 Years: Application of a Questionnaire Established for Adults in Preschool Children
Source: J Epidemiol. 2015 May 5;25(5):341–50. doi: 10.2188/jea.JE20140174 (PMC4411233; doi:10.2188/jea.JE20140174)
Supplement: Abstract in Japanese. [file je-25-341-s001.pdf]

3－6 歳の日本人幼児用簡易型自記式食事歴法質問票 (BDHQ3y) の相対的妥当性と再現性：成人用質問票の就学前児童への応用

朝倉敬子[1,2]、芳賀めぐみ[3]、佐々木敏[2]
[1] 東京大学大学院情報学環、[2] 東京大学大学院医学系研究科　公共健康医学専攻　疫学保健学講座　社会予防疫学分野、[3] 石巻専修大学共創研究センター

**背景**：年少児における食事調査とそれに引き続く食育もしくは食事への介入は、成年後の様々な非感染性疾患を減少させるのに重要である。日本においては、就学前児童を対象とした食事調査用質問票の妥当性評価は始まったばかりである。本研究では、成人においては広範な目的で使用されている簡便な食事調査用質問票である簡易型自記式食事歴法質問票（BDHQ）を 3－6 歳の幼児用に改変（BDHQ3y）し、日本人幼児において BDHQ3y の妥当性の評価を行った。

**方法**：3－4 歳の幼児の保護者 61 人が 1 か月間隔で 2 回、BDHQ3y に回答した。また、その 2 回の調査の間の連続しない 3 日間において、食事記録法による食事調査が行われた。エネルギーと 42 の栄養素の摂取量が、食事記録法と BDHQ3y の両方で推定された。2 つの方法で推定された平均摂取量が比較され、相関係数が計算された。BDHQ3y の推定値の再現性は、級内相関係数（ICC）を用いて検討された。

**結果**：食事記録法と BDHQ3y で推定された平均摂取量は、検討したうちの 1/3～2/3 のエネルギー・栄養素において有意差が認められなかった。残差法でエネルギー調整された摂取量についてのピアソン相関係数の中央値（四分位範囲）は、0.31（0.24-0.38）であった。粗摂取量の ICC の中央値（四分位範囲）は 0.72（0.63-0.76）であった。

**結論**：BDHQ3y は日本人就学前児童の食事調査に適切な質問票となりえるが、その妥当性は現時点では中程度～低いと言える。幼児の食習慣についてさらなる情報を得て質問票に反映させていくことで、欠点を克服してゆく必要がある。

キーワード：妥当性、再現性、食事歴法質問票、日本人、就学前児童
